# Ets2 suppresses inflammatory cytokines through MAPK/NF-κB signaling and directly binds to the IL-6 promoter in macrophages

**DOI:** 10.18632/aging.102480

**Published:** 2019-11-27

**Authors:** Xianwei Ma, Zhengyu Jiang, Na Li, Wei Jiang, Peng Gao, Mingjin Yang, Xiya Yu, Guifang Wang, Yan Zhang

**Affiliations:** 1 Scientific Research Center, Shanghai Public Health Clinical Center, Fudan University, Shanghai 201508, China; 2 Faculty of Anesthesiology, Changhai Hospital, Second Military Medical University/Naval Medical University, Shanghai 200433, China; 3 Department of Respiration, Second Medical Center of Chinese PLA General Hospital, Beijing 100853, China; 4 Cancer Institute, Institute of Translational Medicine, Second Military Medical University/Naval Medical University, Shanghai 200433, China; 5 National Key Laboratory of Medical Immunology and Institute of Immunology, Second Military Medical University/Naval Medical University, Shanghai 200433, China; 6 Department of Respiratory Diseases, Huashan Hospital, Fudan University, Shanghai 200433, China

**Keywords:** Ets2, Toll-like receptor, pro-inflammatory cytokine, macrophage, IL-6

## Abstract

Proper activation of Toll-like receptor (TLR)-mediated signaling and production of proinflammatory cytokines are critical for the initiation of innate immunity, while the specific mechanism maintaining inflammatory homeostasis remains mostly unknown. Here, we show that Ets2 is upregulated following LPS and VSV stimulation. Ets2 knockdown or knockout leads to increased IL-6, TNF-α, and IFN-β production in macrophages. Consistently, Ets2-deficient mice show exacerbated inflammatory cytokine production and are more susceptible to CLP-induced sepsis. Mechanistically, Ets2 inhibits the LPS- and VSV-induced activation of ERK1/2, JNK, p38, and p65. Ets2 also binds to the promoter of IL-6 to inhibit transcription. Collectively, the results of the present study show the negative regulatory role of Ets2 in LPS- and VSV-induced inflammation through the suppression of MAPK/NF-κB signaling, direct binding to the IL-6 promoter and inhibition of transcription.

## INTRODUCTION

Toll-like receptors recognize pathogen components and then activate immune cells to produce inflammatory cytokines [1]. TLR4 and TLR7 are two major TLRs that recognize microbial components during bacterial and viral infections, respectively, and respond to lipopolysaccharide from gram-negative bacteria and single-stranded (ssRNA) from viruses to initiate protective immune responses against pathogens [2]. Upon pathogen recognition, TLR4 and TLR7 both recruit protein MyD88 to activate downstream signal cascades, which cumulate in MAPK and NF-κB pathway activation and induce the production of inflammatory cytokines [2–5]. Proper production of inflammatory cytokines following TLR4 or TLR7 activation is required to initiate innate immunity in defense against pathogens. However, uncontrolled activation and production of inflammatory cytokines may induce tissue damage and lethal endotoxin shock or sepsis. Excessive proinflammatory cytokine production may also be induced by endogenous TLR4 ligands and contribute to chronic inflammation, autoimmune diseases, and cancers [6, 7]. Thus, it is essential to understand the mechanism by which the TLR-activated production of proinflammatory cytokines is regulated.

V-ets erythroblastosis virus E26 oncogene homolog 2 (Ets2) is a member of the Ets transcription factor family. Similar to other members of the family, Ets2 controls the expression of its target genes by binding GGA(A/T) ETS response elements (ERE). Ets2 has been found to impact a broad spectrum of cellular functions, including proliferation, differentiation, migration, transformation, and apoptosis [8, 9]. Research in oncology has also discovered dual functions of Ets2 in controlling cancer proliferation and progression [10, 11]. In studies of inflammation, Ets2 was shown to play a crucial role in persistent activation of TNF-α and increase TNF-α-induced expression of proinflammatory cytokines [12]. Studies have also reported that Ets2 is a target of IL-10 and promotes LPS-induced Mir-155 expression, which further attenuates inflammation and inflammatory cytokine production [13].

In the present study, we demonstrate that Ets2 negatively regulates LPS and vesicular stomatitis virus (VSV)-induced proinflammatory cytokine production in macrophages. We measured cytokine expression and production of IL-6, TNF-α and IFN-β in Ets2 knockdown or knockout macrophages and susceptibility to cecal ligation and puncture (CLP)-induced sepsis in Ets2-deficient mice. We also report direct and indirect mechanisms of Ets2 in regulating the MyD88-dependent inflammatory cascade through MAPK/NF-κB signaling and epigenetic regulation of the IL-6 promoter that attenuates inflammation.

## RESULTS

### LPS or VSV stimulation promotes Ets2 expression and nuclear translocation

**Figure 1:**
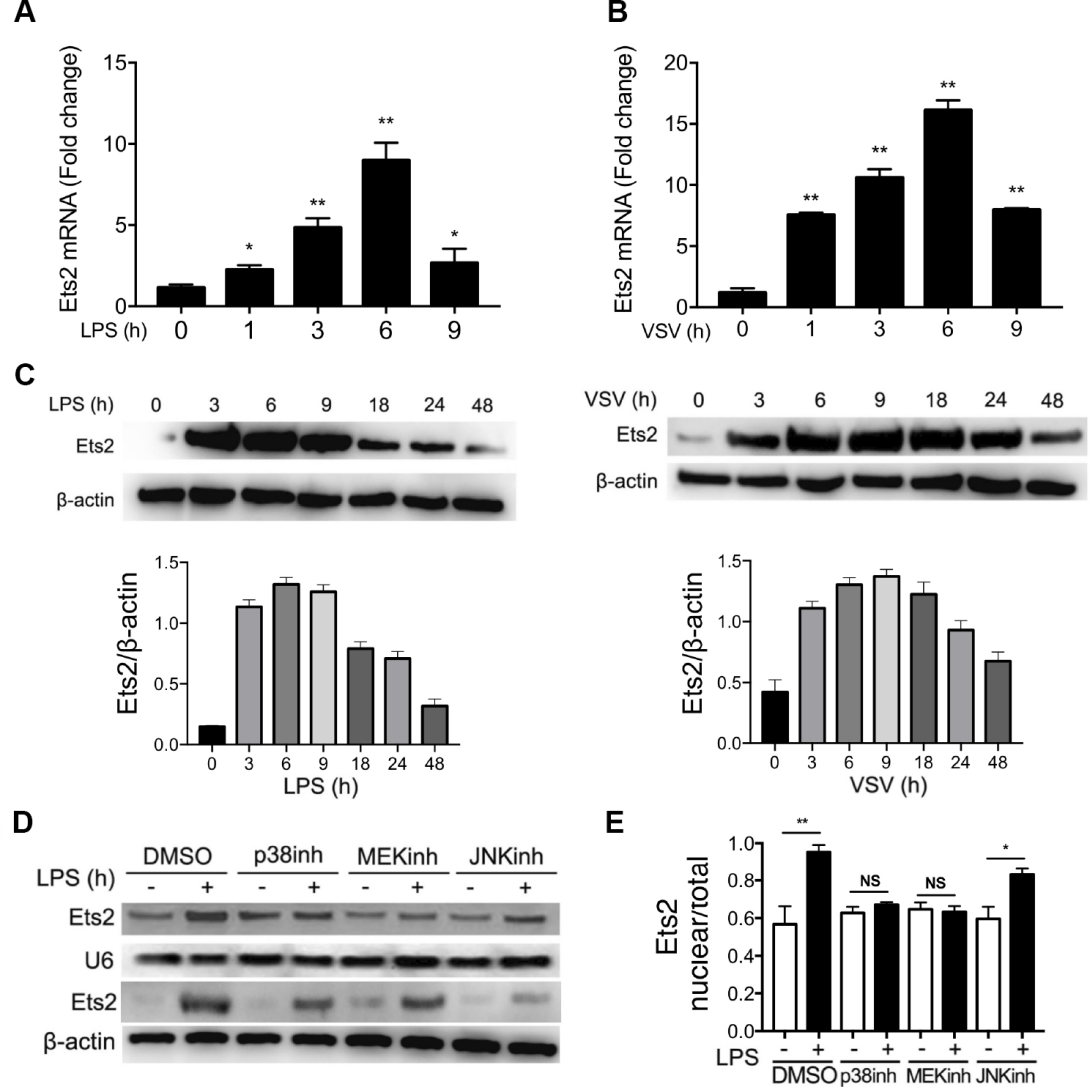
**LPS and VSV promote Ets2 expression and nuclear translocation.** (**A**, **B**) Ets2 mRNA expression in mouse primary peritoneal macrophages stimulated with 100 ng/ml LPS (**A**) or VSV at an MOI of 10 (**B**) for the indicated times, as detected by real-time PCR (n=3). (**C**) Immunoblot analysis and quantification of Ets2 expression in mouse primary peritoneal macrophages stimulated with 100 ng/ml LPS or VSV at an MOI of 10 for the indicated times. (**D**, **E**) Immunoblot analysis (**D**) and quantitative measurement (**E**) of nuclear/total lysate ratio of Ets2 of mouse primary peritoneal macrophages pretreated with SB203580, PD98059, or SP600125 (20 μM for 30 min) and then stimulated with or without 100 ng/ml LPS for 3 h. U6 was used as an internal reference for the nucleus. Data are shown as the mean ± s.d. Student’s t-test compared with the control group. *, P<0.05, and **, P<0.01.

To investigate whether Ets2 could be regulated by the activation of TLR4 and TLR7 signaling, we used mouse primary peritoneal macrophages treated with LPS or VSV to evaluate Ets2 expression. As shown in Figure 1A–1C, the mRNA levels of Ets2 increased and peaked at 6h after stimulation with LPS or VSV, whereas the protein level peaked at 6h and 9h after LPS or VSV stimulation respectively. Because Ets2 translocates into the nucleus to initiate transcription of downstream genes, we asked what signal mediates the translocation of Ets2 in the nucleus. We pretreated cells with the p38 inhibitor SB203580, the MEK inhibitor PD98059, or the JNK inhibitor SP600125 to inhibit LPS-induced p38, ERK1/2, or JNK1/2 activation. The results showed that inhibitors of the ERK1/2 and p38 pathways significantly reduced Ets2 nuclear distribution (Figure 1D, 1E), suggesting that Ets2 was activated through the ERK1/2 and p38 pathways in TLR4 signaling.

### Ets2 negatively regulates LPS- or VSV-stimulated proinflammatory cytokine production

**Figure 2:**
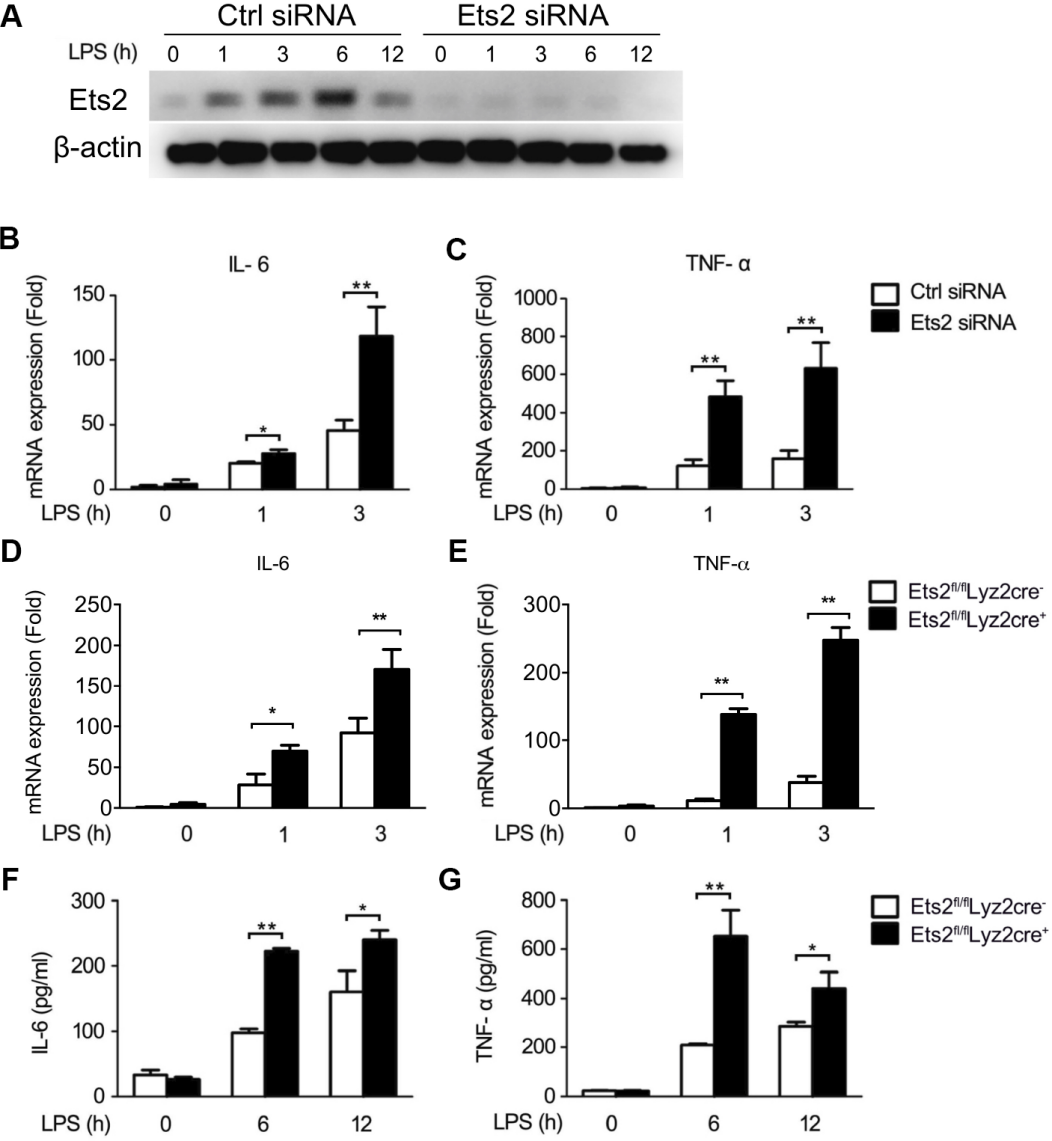
**Ets2 inhibits LPS-induced IL-6 and TNF-α production in macrophages.** (**A**) Immunoblot analysis of the expression of Ets2 in mouse primary peritoneal macrophages transfected with control siRNA (Ctrl) or Ets2-targeted siRNA (Ets2) with LPS stimulation for indicated time. (**B,** **C**) IL-6 (**B**) and TNF-α (**C**) mRNA expression of cells stimulated with 100 ng/ml LPS for the indicated times. (**D**, **E**) IL-6 (**D**) and TNF-α (**E**) mRNA expression in Ets2^fl/fl^Lyz2cre^−^or Ets2^fl/fl^Lyz2cre^+^ mouse primary peritoneal macrophages stimulated with 100 ng/ml LPS for the indicated times. (**F**, **G**) ELISA assay of IL-6 (**F**) and TNF-α (**G**) in the supernatant of the cells stimulated with 100 ng/ml LPS for the indicated times. Data are shown as the mean ± s.d. of three samples. Student’s t-test compared with the control or Ets2^fl/fl^Lyz2cre^-^ group. *, P<0.05, and **, P<0.01.

**Figure 3:**
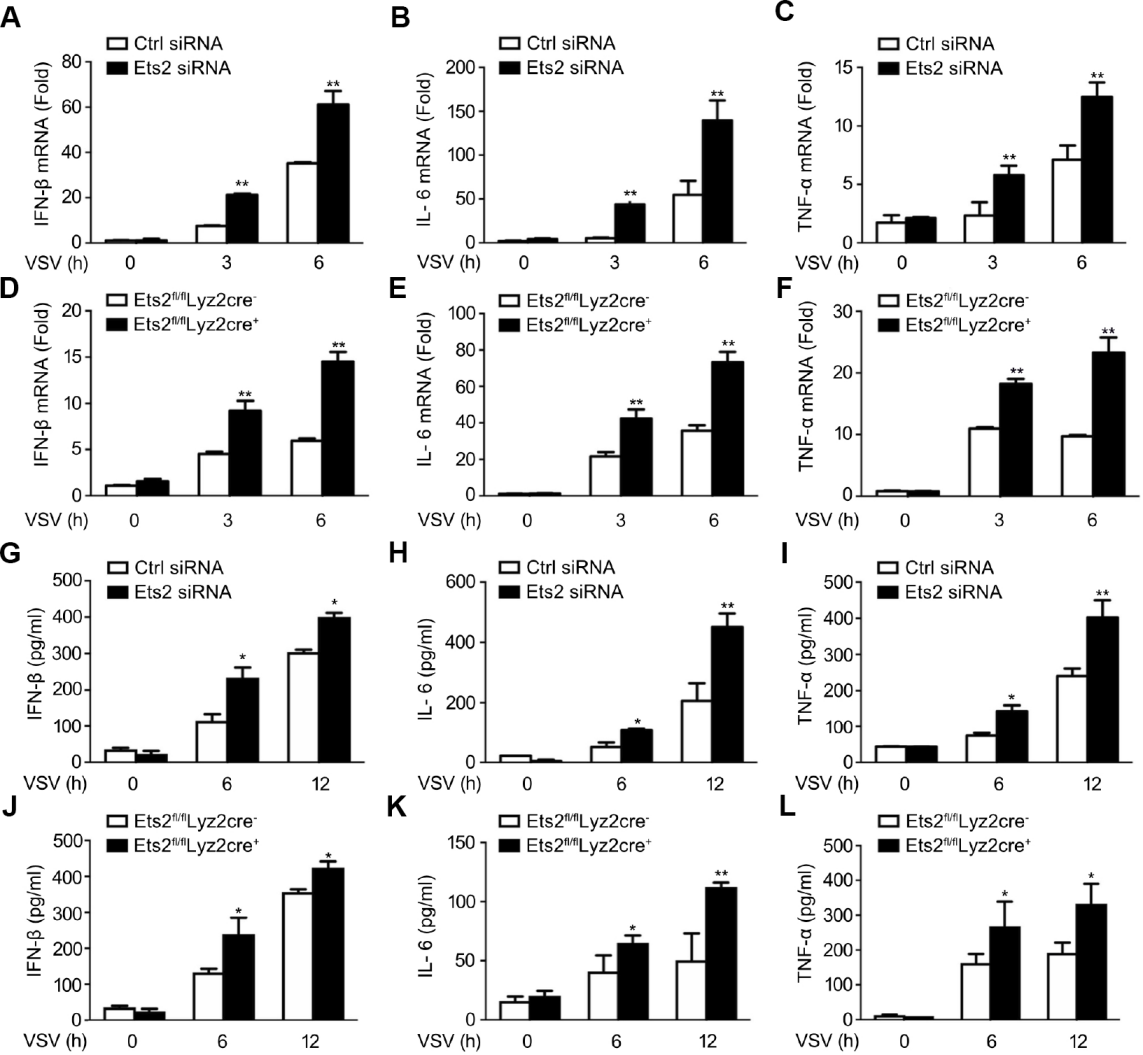
**Ets2 inhibits VSV-induced IFN-β, IL-6, and TNF-α production in macrophages.** (**A**–**C**) IFN-β (**A**), IL-6 (**B**) and TNF-α (**C**) mRNA expression of cells stimulated with VSV at an MOI of 10 for the indicated times. d-f: IFN-β (**D**), IL-6 (**E**), and TNF-α (**F**) mRNA expression in Ets2^fl/fl^Lyz2cre^−^ or Ets2^fl/fl^Lyz2cre^+^ mouse primary peritoneal macrophages stimulated with VSV at an MOI of 10 for the indicated times. (**G**–**I**) ELISA assay of IFN-β (**G**), IL-6 (**H**), and TNF-α (**I**) in the supernatants of cells stimulated with VSV at an MOI of 10 for the indicated times. (**J**–**L**) ELISA assay of IFN-β (**J**), IL-6 (**K**), and TNF-α (**L**) in the supernatants of the cells stimulated with VSV at an MOI of 10 for the indicated times in Ets2^fl/fl^Lyz2cre^−^or Ets2^fl/fl^Lyz2cre^+^ mouse primary peritoneal macrophages. Data are shown as the mean ± s.d. of three samples. Student’s t-test compared with the control or Ets2^fl/fl^Lyz2cre^-^ group. *, P<0.05, and **, P<0.01.

We then investigated the possible role of Ets2 in LPS and VSV responses. We first demonstrated that the small interfering RNA specific to Ets2 (Ets2 siRNA) efficiently inhibited endogenous Ets2 expression within 12h after LPS stimulation in mouse peritoneal macrophages (Figure 2A). Afterward, interference of Ets2 significantly increased LPS-induced IL-6 and TNF-α mRNA expression in the cells (Figure 2B, 2C), suggesting that Ets2 might negatively regulate LPS-induced IL-6 and TNF-α mRNA expression. To further confirm the role of Ets2 in LPS-induced IL-6 and TNF-α mRNA expression, we generated conditional Ets2-deficient mice by crossing Ets2flox mice with Lyz2-Cre mice. Ets2-deficient mouse primary peritoneal macrophages (Ets2^fl/fl^Lyz2cre^+^) and control cells (Ets2^fl/fl^Lyz2cre^−^) were prepared. As shown in Figure 2D–2G, Ets2 deficiency significantly increased LPS-induced IL-6 and TNF-α mRNA expression (Figure 2C, 2D) and production (Figure 2F, 2G) in mouse peritoneal macrophages. A similar phenomenon was also observed in VSV-stimulated Ets2 siRNA or conditional knockout macrophages. The mRNA level (Figure 3A–3F) and cytokine production (Figure 3G–3L) were all elevated when Ets2 was knocked out or knocked down. These results demonstrated that Ets2 functioned as a negative regulator of LPS- or VSV-induced proinflammatory cytokine production in mouse primary peritoneal macrophages.

### Ets2 suppresses LPS-induced inflammation and improves mouse survival against sepsis

**Figure 4:**
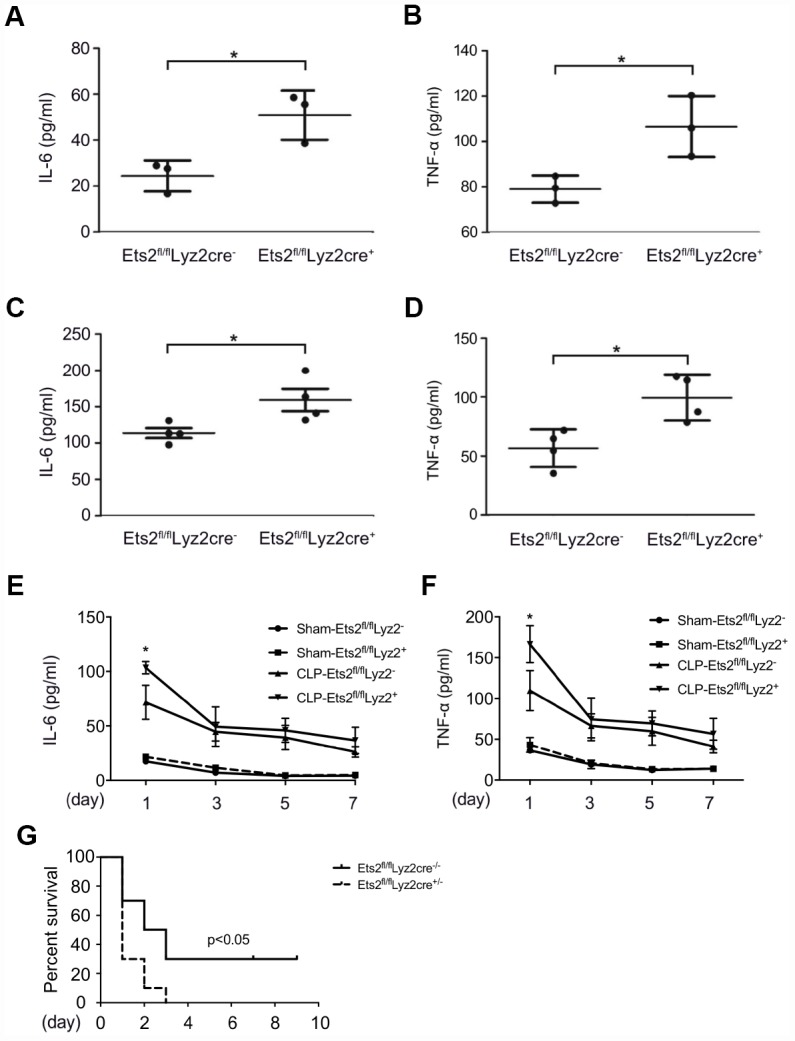
**Ets2 inhibits LPS-induced IL-6 and TNF-α production and improves the survival of CLP-induced sepsis *in vivo*.** (**A**, **B**) ELISA assay of IL-6 (**A**) and TNF-α (**B**) in the serum of Ets2^fl/fl^Lyz2cre^−^ or Ets2^fl/fl^Lyz2cre^+^ mice, intraperitoneally injected with LPS (1 μg/g body weight) for 5 h (n=3 per phenotype). (**C**, **D**) ELISA assay of IL-6 (**C**) and TNF-α (**D**) in the serum of Ets2^fl/fl^Lyz2cre^−^ or Ets2^fl/fl^Lyz2cre^+^ mice at 6 h after CLP surgery (n=4 per phenotype). (**E**–**G**) Continuous IL-6 (**E**) and TNF-α (**F**) at postoperative day 1, 3, 5, 7, and survival rate (**G**) of Ets2^fl/fl^Lyz2cre^−^ or Ets2^fl/fl^Lyz2cre^+^ mice given CLP or Sham surgery (n=10 per phenotype). Data are shown as the mean ± s.e.m. Student’s t-test compared with the Ets2^fl/fl^Lyz2cre^-^ group for ELISA experiments. A log-rank test was used for survival data. *, P<0.05.

After demonstrating the negative regulation of Ets2 on proinflammatory cytokine production, we asked whether Ets2 plays a protective role in mouse endotoxemia or sepsis. We first found that compared to wild-type mice, Ets2-deficient mice showed higher levels of IL-6 and TNF-α after LPS challenge (Figure 4A, 4B), indicating the crucial role of Ets2 in attenuating inflammation in mouse endotoxemia. Moreover, in severe sepsis model (cecum ligated for 1cm in CLP procedure), Ets2-deficient mice also showed increased IL-6 and TNF-α production in serum (Figure 4C–4D) and poor survival (Figure 4G). Furthermore, in the mild sepsis model (cecum ligated for 0.5cm in CLP procedure), Ets2-deficient mice also showed elevated IL-6 and TNF-α production in serum while showed no difference at day 3, 5 and 7 (Figure 4E, 4F). Organ damage showed little significance among two groups (Supplementary Figure 3). These results indicated the protective effects of Ets2 in a mouse endotoxemia and sepsis model via the suppression of inflammation *in vivo*.

### Ets2 negatively regulates MAPK/NF-κB signaling in macrophages

**Figure 5:**
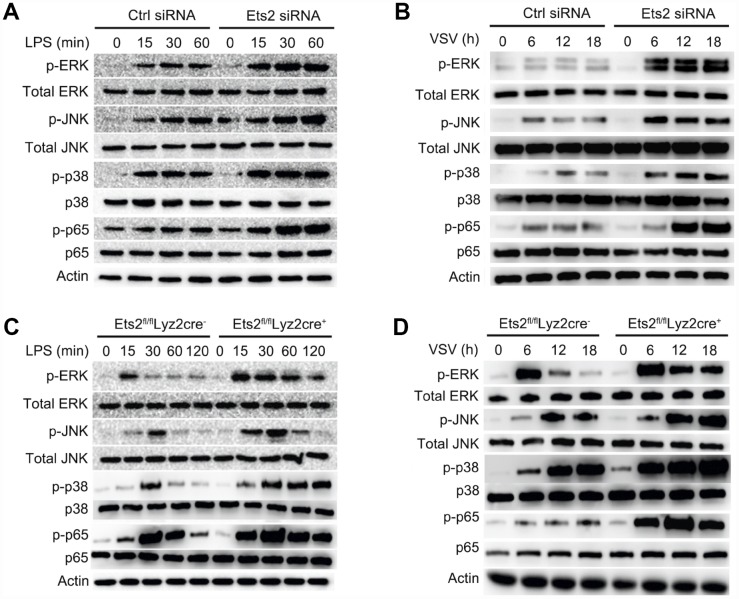
**Ets2 inhibits NF-κB and MAPK signaling.** (**A**, **B**) Immunoblot analysis of phosphorylated and total ERK1/2, JNK, p38 and NF-κB p65 in mouse primary peritoneal macrophages transfected with control siRNA (Ctrl siRNA) or Ets2-targeted siRNA (Ets2 siRNA) (**A**) and primary peritoneal macrophages of Ets2^fl/fl^Lyz2cre^−^ or Ets2^fl/fl^Lyz2cre^+^ mice (**B**), stimulated with 100 ng/ml LPS for the indicated times. (**C**, **D**) Immunoblot analysis of phosphorylated and total ERK1/2, JNK, p38, and NF-κB p65 in primary peritoneal macrophages transfected with control siRNA (Ctrl siRNA) or Ets2-targeted siRNA (Ets2 siRNA) (**C**) and primary peritoneal macrophages of Ets2^fl/fl^Lyz2cre^−^ or Ets2^fl/fl^Lyz2cre^+^ mice (**D**) stimulated with VSV at an MOI of 10 for the indicated times.

Proinflammatory cytokine production relies on the transduction of multiple signals, which are predominantly regulated by the activation of NF-κB and MAPK signaling [2, 14]. To investigate whether Ets2 affects the activation of these signaling pathways, we used Ets2 knockdown primary peritoneal macrophages or cells from knockout mice to evaluate the effects on molecules in the signaling pathway. Ets2 siRNA -treated or Ets2-deficient macrophages all showed elevated phosphorylation of NF-κB and MAPK signaling components (Figure 5A–5D). Particularly in Ets2-deficient PMs, p-ERK, p-JNK, p-p38, and p-p65 were all elevated by LPS (Figure 5C) or VSV (Figure 5D) stimulation. Thus, these results suggest that Ets2 may suppress inflammation by negatively regulating MAPK/NF-κB signaling.

### Ets2 inhibits IL-6 transcription by binding to the IL-6 promoter

**Figure 6:**
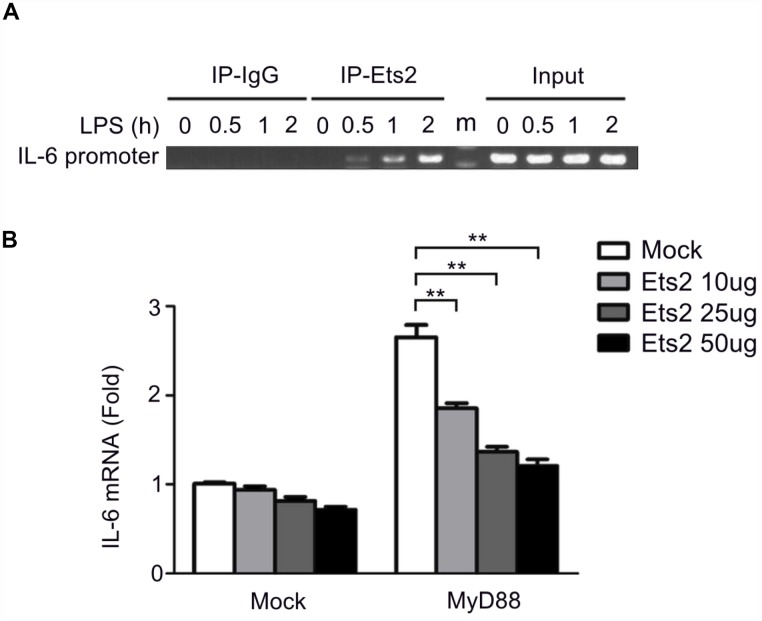
**Ets2 inhibits IL-6 transcription by binding to the IL-6 promoter.** (**A**) ChIP analysis of the IL-6 promoter using an Ets2 antibody in mouse primary peritoneal macrophages treated with LPS for the indicated times. (**B**) Luciferase assay of IL-6 reporter gene expression in HEK293 cells transfected with 50 ng of a MyD88-expressing plasmid, 90 ng of an IL-6 luciferase reporter plasmid, and 10 ng of a pTK-Renilla-luciferase reporter plasmid, together with 0, 10, 25 or 50 ng of an Ets2-expressing plasmid. Luciferase activity was measured and normalized to Renilla luciferase activity. Data are shown as the mean ± s.d. Student’s t-test compared with the control group. **, P<0.01.

Previous studies proposed that Ets2 functions by binding to the promoter of interleukin genes in T cells [15]. We asked whether Ets2 has a similar mechanism in regulating cytokines in macrophages. We checked the recruitment of Ets2 to the IL-6 promoter following LPS stimulation by chromatin immunoprecipitation (ChIP) assay. As shown in Figure 6A, LPS stimulation significantly increased the recruitment of Ets2 to the IL-6 promoter. IL-6 transcription is dependent on MyD88 in LPS-stimulated signaling. Thus, we tested the effects of MyD88 and Ets2 overexpression on IL-6 promoter-driven luciferase reporter gene expression in HEK293 cells. As expected, overexpression of MyD88 increased IL-6 reporter gene expression (Figure 6B). Moreover, overexpression of Ets2 alone did not significantly affect the expression of the IL-6 reporter gene, whereas, in the presence of MyD88, Ets2 significantly inhibited IL-6 reporter gene expression. These results indicate that Ets2 inhibits MyD88-induced IL-6 transcription.

**Figure 7:**
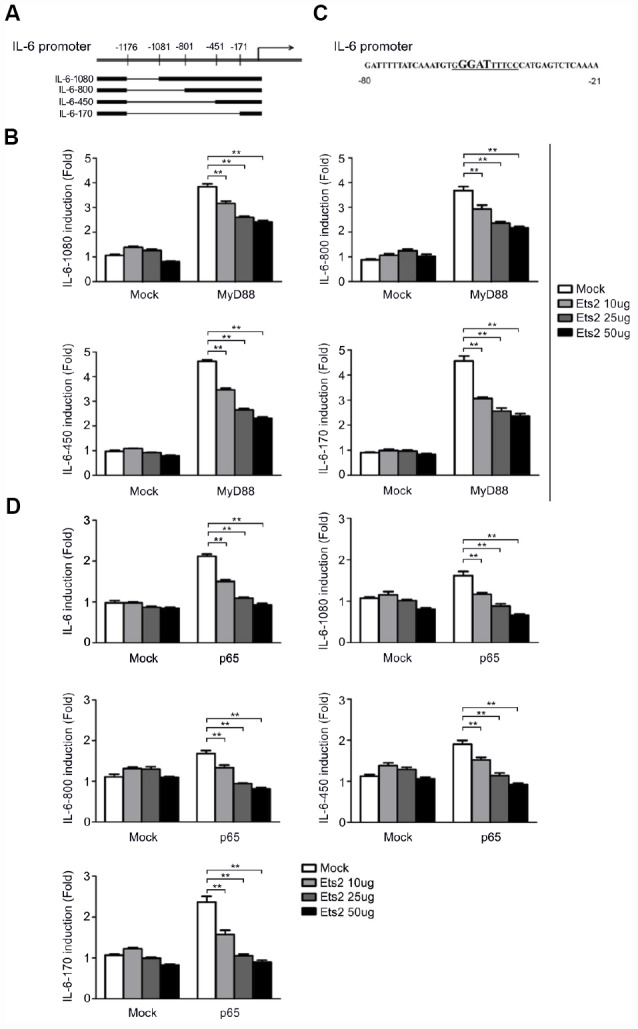
**Ets2 inhibits NF-κB-dependent IL-6 transcription.** (**A**) Schematic diagram of truncated IL-6 promoter-reporter plasmids constructed by deleting the regions at −1176/−1081, −1176/−801, −1176/−451, and −1176/−171. (**B**) Luciferase assay in HEK293 cells transfected with 50 ng of a MyD88-expressing plasmid, 90 ng of the truncated IL-6 promoter-luciferase reporter plasmids, and 10 ng of a pTK-Renilla-luciferase reporter plasmid, together with 0, 10, 25 or 50 ng of an Ets2-expressing plasmid. Luciferase activity was measured and normalized by Renilla luciferase activity. (**C**) IL-6 promoter sequence shown from position -80 to -21. (**D**) Luciferase assay in HEK293 cells transfected with 50 ng of a p65-expressing plasmid, 90 ng of the truncated IL-6 luciferase reporter plasmids, and 10 ng of a pTK-Renilla-luciferase reporter plasmid, together with 0, 10, 25 or 50 ng of an Ets2-expressing plasmid. Luciferase activity was measured and normalized to Renilla luciferase activity. Data are shown as the mean ± s.d. Student’s t-test compared with the control group. **, P<0.01.

We then constructed truncated IL-6 promoter-reporter plasmids by deleting the regions at −1176/−1081, −1176/−801, −1176/−451, and −1176/−171 to localize the functional Ets2 response element in the IL-6 promoter (Figure 7A). As shown in Figure 7B, all of the deletions did not abolish Ets2-mediated negative regulation of MyD88-induced reporter gene expression, suggesting the binding site of Ets2 starts from −170 of the IL-6 promoter to regulate reporter gene expression. Analysis of the IL-6 promoter DNA sequence revealed a putative NF-κB binding site, GGGATTTTCC, in the Ets2-binding region (Figure 7C). Interestingly, the GGGATTTTCC sequence also contains a GGAT Ets consensus sequence. As shown in Figure 7D, Ets2 inhibited NF-κB p65 overexpression-induced IL-6 reporter gene expression. It is reasonable to assume that Ets2 might negatively regulate IL-6 production by inhibiting NF-κB-mediated IL-6 gene transcription.

## DISCUSSION

Inflammation occurs in multiple aging-related diseases including hypertension, diabetes, cancer and frailty [16, 17]. Investigating regulators in immune homeostasis may help to understand the pathogenesis and related mechanism in aging process or geriatric diseases, which includes the TLR-mediated innate immunity responses [17]. Regulation of TLR signaling and the downstream cascades can divide host responses to cause protective inflammation against pathogens or lethal endotoxemia and sepsis. Transcription factors show different functions in regulating inflammation, for example, AP-1, IRF5, or Fil-1 increase proinflammatory cytokines in most inflammatory models, whereas c-Fos suppresses systematic inflammation to endotoxin [14, 18–21]. Identifying key regulators and mechanisms in inflammatory responses provides supplemental knowledge and indications for translational application in clinical inflammatory diseases. In the present study, we found that Ets2 was increased and translocated into the nucleus through ERK1/2 and p38 activation following LPS stimulation and further suppressed cytokine production that acts as a feedback loop for inflammation regulation. LPS and VSV stimulation activate TLR 4 and TLR 7 respectively and subsequent signaling. Previous studies proposed that Ets2 act as downstream of RAS/RAF/MEK pathway [22], which is consistent with the present finding. Inhibition of ERK1/2 and p38 both attenuated Ets2 expression, suggesting these molecules may act as crucial regulator in Ets2 expression. However, specific epigenetic regulation is still needed to unveil detailed mechanism of Ets2 regulation.

Ets2 deficiency led to exacerbated cytokine production, which is independent of the ERK1/2, p38, and JNK signal as the inhibition of these signals did not reverse the elevation of cytokine production in Ets2-deficient PMs (Supplementary Figures 4, 5). Moreover, Ets2 knockout mice were more susceptible than wild-type mice to CLP-induced sepsis. These results indicate the regulatory role of Ets2 in LPS- and VSV-induced inflammation. Previous studies found that functional Ets2 is required for persistent TNF-α production, while the mutant allele decreases the expression of TNF-α [23]. However, Guo Wei et al. [23] used viable motheaten mutant mice to investigate the function of Ets2, in which Ets2 was constitutively activated by the PI3K/Jun N-terminal kinase pathway, whereas in wild-type mice, Ets2 was activated by cytokines such as CSF-1. Moreover, the role of Ets2 in TNF-α production was based on a chronic inflammation scenario [23, 24]. Therefore, the present study offers a new perspective that Ets2 exerts anti-inflammatory effects in acute inflammation, which indicates the diverse function of Ets2 in the regulation of inflammation.

LPS and VSV are thought to activate TLR4 and TLR7, respectively, to initiate downstream inflammatory cascades [2, 3]. Similar to LPS-induced TLR4 activation, VSV stimulation also recruits MyD88 to activate the downstream NF-κB and MAPK pathways. IFN-β, a type I interferon-stimulated by TLR7 in virus infection [4], was measured to monitor the effects of Ets2 in VSV treatment. Therefore, in the present study, LPS and VSV treatment increased the effects of Ets2 on the TLR-mediated activation of the MyD88-MAPK/NF-κB pathway, which provides broader evidence of Ets2 in regulating inflammation via a mechanism similar to that of TLR 4 and 7. However, it should also be noted that VSV stimulation may also be MyD88-independent, which alternatively acts via TRAF2 and TRAF3 to induce type I interferon and antiviral immunity [25, 26]. Therefore, further studies are needed to investigate the possible role of Ets2 in the MyD88-independent mechanism of inflammatory regulation.

Consistent with the increased cytokine production in LPS- and VSV-stimulated macrophages increased activation of ERK1/2, JNK, p38, and p65 was found in the Ets2 knockdown and knockout model in the present study, which suggested that the downregulation of MAPK/NF-κB signaling may be one mechanism in the regulation of cytokine production. In addition, by analyzing the regulation of IL-6 expression, we found that Ets2 could directly bind to the IL-6 promoter and inhibit its transcription. Ets2 overexpression inhibited MyD88-induced IL-6 promoter-reporter gene expression. These results demonstrated that Ets2 inhibited LPS-induced IL-6 production partially by directly binding to the IL-6 promoter and regulating IL-6 gene transcription. Deletion of the 5' region of the IL-6 promoter did not affect Ets2-mediated negative regulation of IL-6 promoter-reporter gene expression, suggesting that Ets2 regulated IL-6 gene transcription by binding to the proximal region of the IL-6 promoter. Interestingly, there is a putative NF-κB binding site (GGGATTTTCC) in the proximal promoter region, which also contains an Ets consensus sequence (GGAT). Furthermore, Ets2 overexpression inhibited NF-κB-induced IL-6 promoter-reporter gene expression. According to these results, we speculated that Ets2 inhibited IL-6 production by antagonizing the transcriptional activity of NF-κB that regulates IL-6 gene expression. However, this finding was not reproducible in the regulation of TNF-α, as in the ChIP analysis of TNF-α, we did not observe the binding or regulation of Ets2 in the promoter region of TNF-α (Supplementary Figure 6). Therefore, the regulation of TNF-α expression by Ets2 might be the result of the suppression of TLR signaling, while the suppression of signaling and direct binding to the promoter may both be reasons for the attenuation of IL-6 production. Thus, the present study proposes a diverse mechanism of Ets2 regulation of proinflammatory cytokine production.

Conclusively, our results indicate the crucial role of Ets2 in regulating inflammatory responses stimulated by LPS and VSV. Ets2 negatively regulates NF-κB and MAPK signaling and directly binds to the IL-6 promoter to inhibit transcription. These findings provide a better understanding of Ets2 in TLR-mediated inflammation and provide new insight into the mechanism by which TLR-mediated innate immunity is regulated.

## MATERIALS AND METHODS

### Mice

Ets2 flox mice were purchased from the Jackson Laboratory and mated with Lyz2-Cre mice. Sex- and age-matched 6–8-week-old Ets2^fl/fl^Lyz2Cre^+^ (Ets2-CKO) mice and littermate Ets2^fl/fl^Lyz2Cre^−^ (Ets2-WT) mice were used. C57BL/6 mice (6-8 weeks old) were obtained from Joint Ventures Sipper BK Experimental Animal Company (Shanghai, China). The present study was conducted in accordance with the National Institute of Health Guide for the Care and Use of Laboratory Animals and was also reviewed and approved by the Animal Experimental Ethical Inspection of Laboratory Animal Centre, Second Military Medical University.

### Reagents and cell culture

LPS was obtained from Sigma, USA (437628). The pharmacological inhibitors SB203580, PD98059, and SP600125, were obtained from Selleck Chemicals, USA. Antibodies specific for β-actin (sc-1616), Ets2 (sc-351), p38 (sc-535), and ERK1/2 (sc-93) were from Santa Cruz Biotechnology, USA. Antibodies specific for p65 (4764), JNK (9052), phosphorylated ERK1/2 (9101), phosphorylated p38 (9216), phosphorylated p65 (3033) and phosphorylated JNK (9251) were from Cell Signaling Technology, USA. The antibody specific for β-actin was applied at a dilution of 1:5,000. All other antibodies were applied at a dilution of 1:1,000. The HEK293 cell line was obtained from American Type Culture Collection, USA, and maintained in DMEM (Hyclone, USA) with 10% (vol/vol) FBS (Gibco, USA).

### Peritoneal macrophage preparation

Primary peritoneal macrophages (PMs) of C57BL/6 or conditional Ets2-deficient mice (6–8 weeks old) were prepared by intraperitoneal injection with 2 ml of 3% sodium thioglycollate (Merck, USA) solution for three days as previously reported [27]. Generation of PMs by this method has been regarded as one of the methods in generating mouse primary macrophages [28] with verification by flow cytometry marked by CD11b and F4/80, the marker of macrophage (Supplementary Figure 1). Ets2 expression in CD11b+/ F4/80+ cells generated from Ets2-deficient mice (Ets2f^l/fl^Lyz2Cre^+^) was verified in Supplementary Figure 2. Cells collected in the abdominal cavity were seeded and maintained in DMEM with 10% (vol/vol) FBS for further experiments.

### RNA interference

Mouse primary peritoneal macrophages were transfected with siRNA (30 nM) using INTERFERin reagent (Polyplus Transfection, Thermo, USA). The specific On-target siRNA for Ets2 (L-040983-01, Thermo, USA) and Non-target siRNA for Ctrl were obtained from Thermo, USA.

### Real-time-quantitative PCR

Total RNA was extracted using an RNA extraction kit, purchased from Fastagen Biotech (Shanghai, China), and reverse-transcribed using reverse transcriptase M-MLV (Takara, Japan). Reverse transcription products were amplified by ABI7500 (Life Tech, USA) using a qPCR Master Mix (Promega, USA). Data were normalized to the level of β-actin in each sample. The primers used were as follows: mouse β-actin, 5′ AGTGTGACGTTGACATCCGT 3′ (forward) and 5′ GCAGCTCAGTAACAGTCCGC 3′ (reverse); mouse Ets2, 5′ CCTGTCGCCAACAGTTTTCG 3′ (forward) and 5′ TGGAGTGTCTGATCTTCACTGA 3′ (reverse); mouse TNF-α, 5′ AAGCCTGTAGCCCACGTCGTA 3′ (forward) and 5′ GGCACCACTAGTTGGTTGTCTTT G 3′ (reverse); and mouse IL-6, 5′-TAGTCCTTCCTAC CCCAATTTCC 3′ (forward) and 5′ TTGGTCCTTAG CCACTCCTTC 3′ (reverse).

### Detection of cytokine production

An ELISA detecting the concentrations of IFN-β, IL-6, and TNF-α in supernatants or serum was performed using ELISA kits from eBioscience (Invitrogen, USA) according to the manufacturer’s instructions.

### Plasmid constructs and luciferase reporter gene expression assay

The IL-6 luciferase reporter plasmid was constructed based on the pGL3 Luciferase Reporter Vector (Promega, USA). Truncated IL-6 promoter-reporter plasmids were constructed by deleting the regions at −1176/−1081, −1176/−801, −1176/−451, and −1176/−171 of the IL-6 promoter. The PRL-TK-Renilla-luciferase plasmid was purchased from Promega, USA. HEK293 cells were cotransfected with a mixture of plasmids, as indicated. Twenty-four hours later, luciferase activity in the cell lysates was measured with a Dual-Luciferase Reporter Assay System (Promega, USA) and normalized to Renilla luciferase activity in each sample.

### Hematoxylin-eosin (HE) staining

Mice underwent CLP surgery was executed 2 or 4 days after the procedure. The liver, lung, and kidney tissues were collected and were fixed in 10% formalin. The fixed specimens were processed to paraffin blocks, sectioned (5 *μ*m), and stained with hematoxylin-eosin (H & E) for histological analysis according to our previous reported protocols [29].

### Chromatin immunoprecipitation (chip) assay

ChIP assays were conducted according to the protocol of the ChIP Assay Kit (Merck Millipore, USA). The region of the mouse IL-6 promoters was amplified with specific primers, 5′-TACCACTCCCAACAGACCTG-3′ (forward) and 5′-GGTACTCCAGAAGACCAGAGG -3′ (reverse); and TNF-α: 5′-CCAAGGGTGGAGAGA GATGAG-3′ (forward) and 5′-GCCTCTGCCATATC TTGACTG-3′ (reverse).

### Immunoblot analysis

The method is as previously described [29]. Cells were lysed on ice with M-PER Protein Extraction Reagent (Pierce, USA) supplemented with a protease inhibitor cocktail and measured with a BCA Protein Assay Kit (Pierce, USA). Nucleus protein was prepared with NE-PER™ Nuclear and Cytoplasmic Extraction Reagents (Thermo Scientific, USA). Equal amounts of protein were subjected to SDS-PAGE, followed by Western blot with the indicated antibodies. Finally, the signal intensity was determined using a Chemiluminescent Imaging System 5200S (Tanon, China).

### Establishment of LPS challenged and CLP-induced sepsis mouse model

Mice (6–8 weeks old) were challenged with LPS intraperitoneally (1 μg of LPS per gram of mouse body weight). Five hours later, the serum was obtained for ELISA assays. Cecal ligation and puncture (CLP) experiments of mice (6–8 weeks old) were performed as described^9^. Mice were anesthetized by inhaling sevoflurane mixed with oxygen via anesthetic evaporator VP300 (Yi’an) during the surgery. Laparotomy was performed with an incision of 1 cm. The cecum was exteriorized and ligated and then punctured with a 22-G needle at the end position. For survival analysis, the cecum was ligated for 1cm, whereas for in vivo cytokine analysis after CLP, the cecum was ligated for 0.5cm. After suturing, mice were monitored for nine days, or blood was drawn six h later for further experiments. For continuous cytokine measurement after CLP procedure, mice were anesthetized, and the capillary tube was used to collect the blood from the post-glomus venous plexus of mice at postoperative day 1, 3, 5 and 7. 50ul blood was collected, and serum was isolated for the ELISA experiments.

### Statistical analysis

The two-tailed unpaired Student’s t-test or the log-rank test (survival curve comparison) was performed using Prism software. P values less than 0.05 were considered significant.

## Supplementary Material

Supplementary Figures
