# Supplementary material for: Ets2 suppresses inflammatory cytokines through MAPK/NF-κB signaling and directly binds to the IL-6 promoter in macrophages
Source: Aging (Albany NY). 2019 Nov 27;11(22):10610–25. doi: 10.18632/aging.102480 (PMC6914388; doi:10.18632/aging.102480)
Supplement: Supplementary Figures [file aging-11-102480-s001..pdf]

SUPPLEMENTARY FIGURES

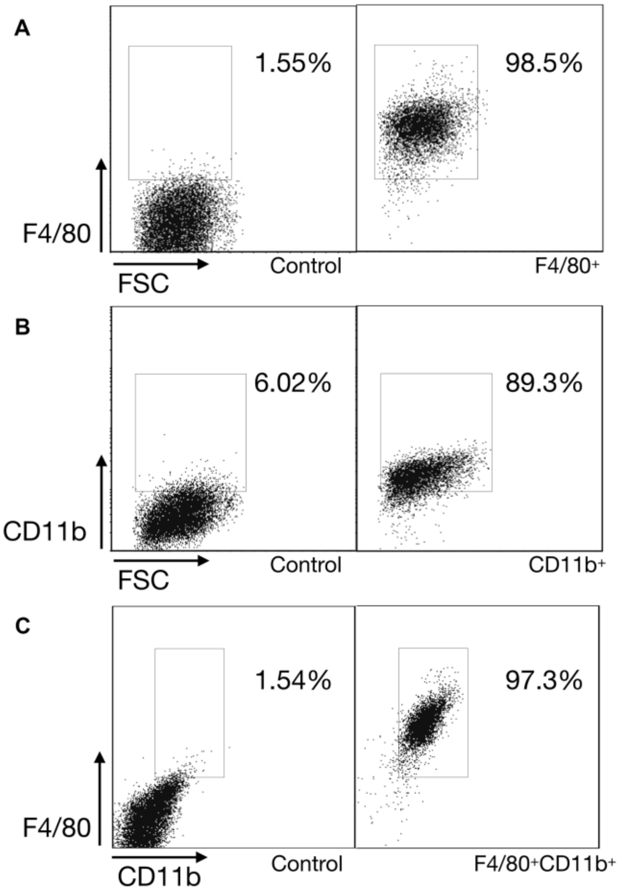

Supplementary Figure 1. Representatives of dot plots of peritoneal macrophage stained with F4/80 and CD11b in flow cytometry.

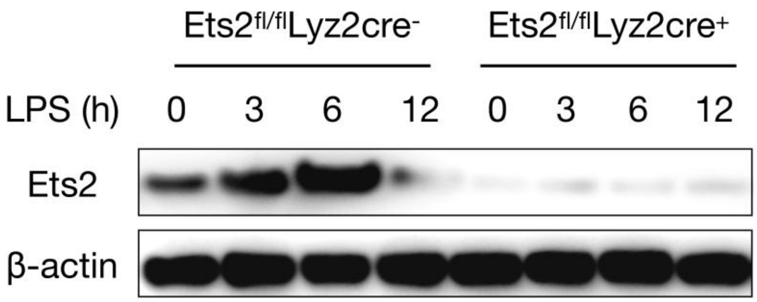

Supplementary Figure 2. Western blot of Ets2 and β-actin of Ets2-deficient (Ets2$^{fl/fl}$Lyz2cre^{+}) or wild-type (Ets2$^{fl/fl}$Lyz2cre^{-}) PMs stimulated with 100 ng/ml LPS in indicated times (0, 3, 6, 12 hours).

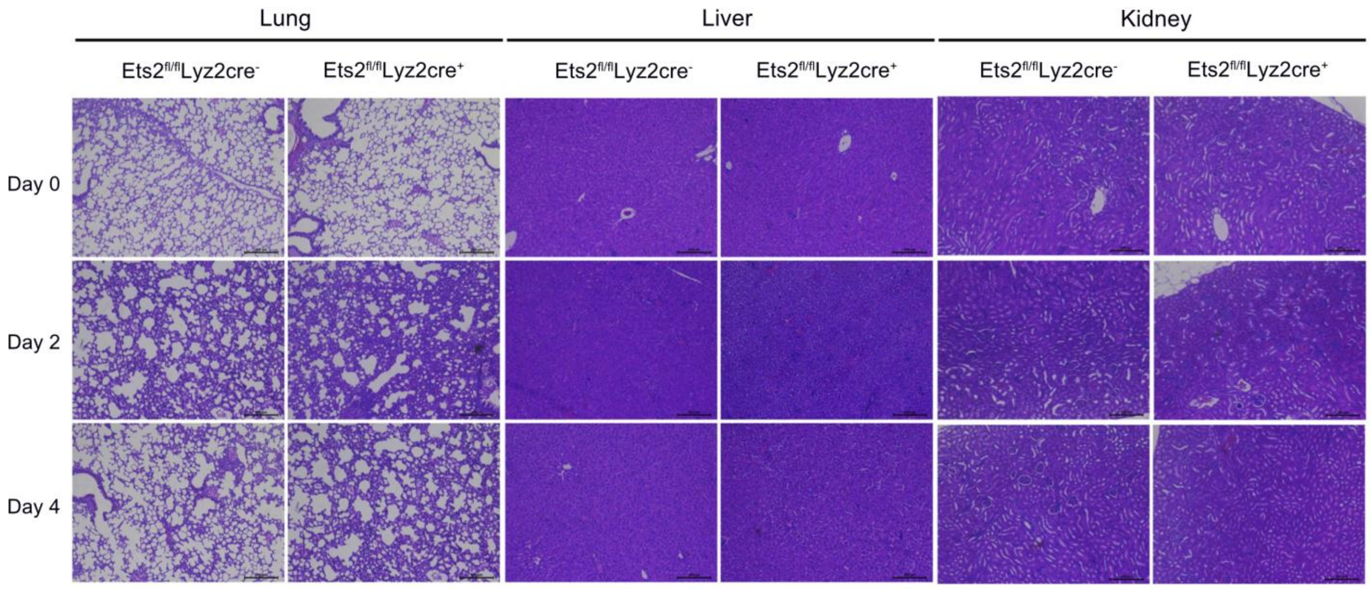

Supplementary Figure 3. Representatives of H&E staining of lung, liver and kidney in postoperative Day 2, and 4 of Ets2$^{fl/fl}$Lyz2cre^{-} or Ets2$^{fl/fl}$Lyz2cre^{+} mice underwent CLP surgery. Day 0 present the Sham surgery procedure.

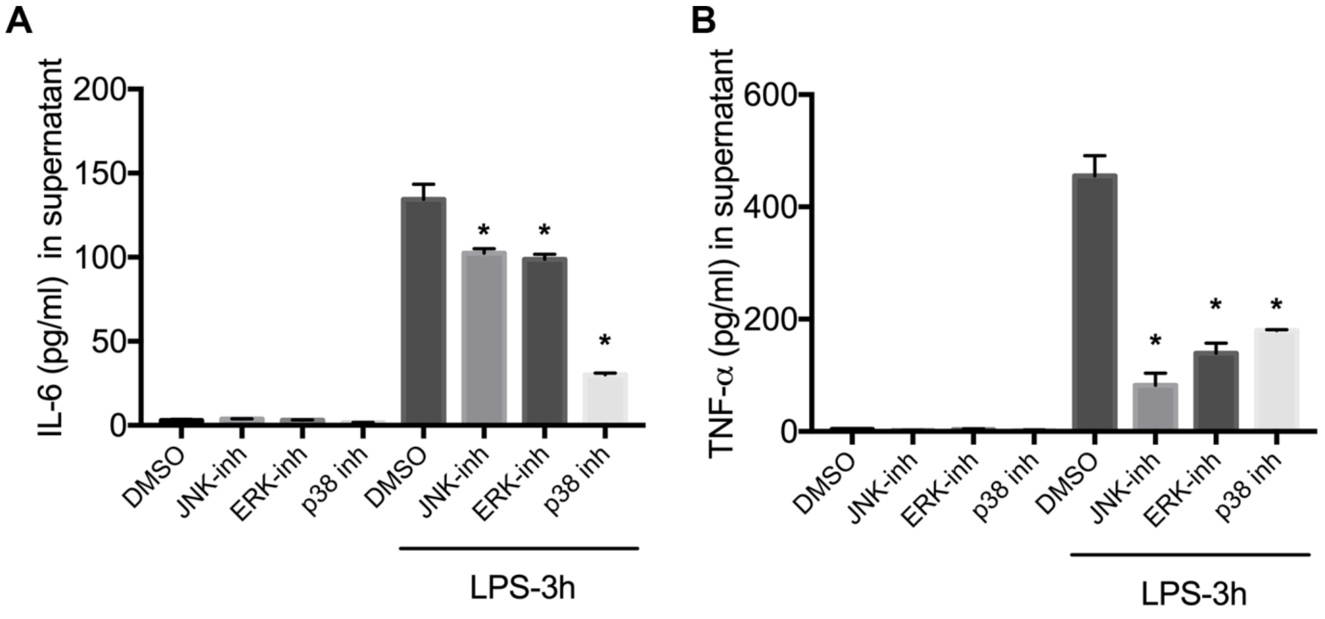

Supplementary Figure 4. (**A**, **B**) ELISA of IL-6 (**A**) and TNF-α in the supernatant of peritoneal macrophage pre-treated with DMSO or inhibitor of ERK, JNK, and p38 respectively and stimulated with LPS for 3h. Data are shown as the mean ± s.d. of three samples. Student's t-test compared with the DMSO. *, P<0.05.

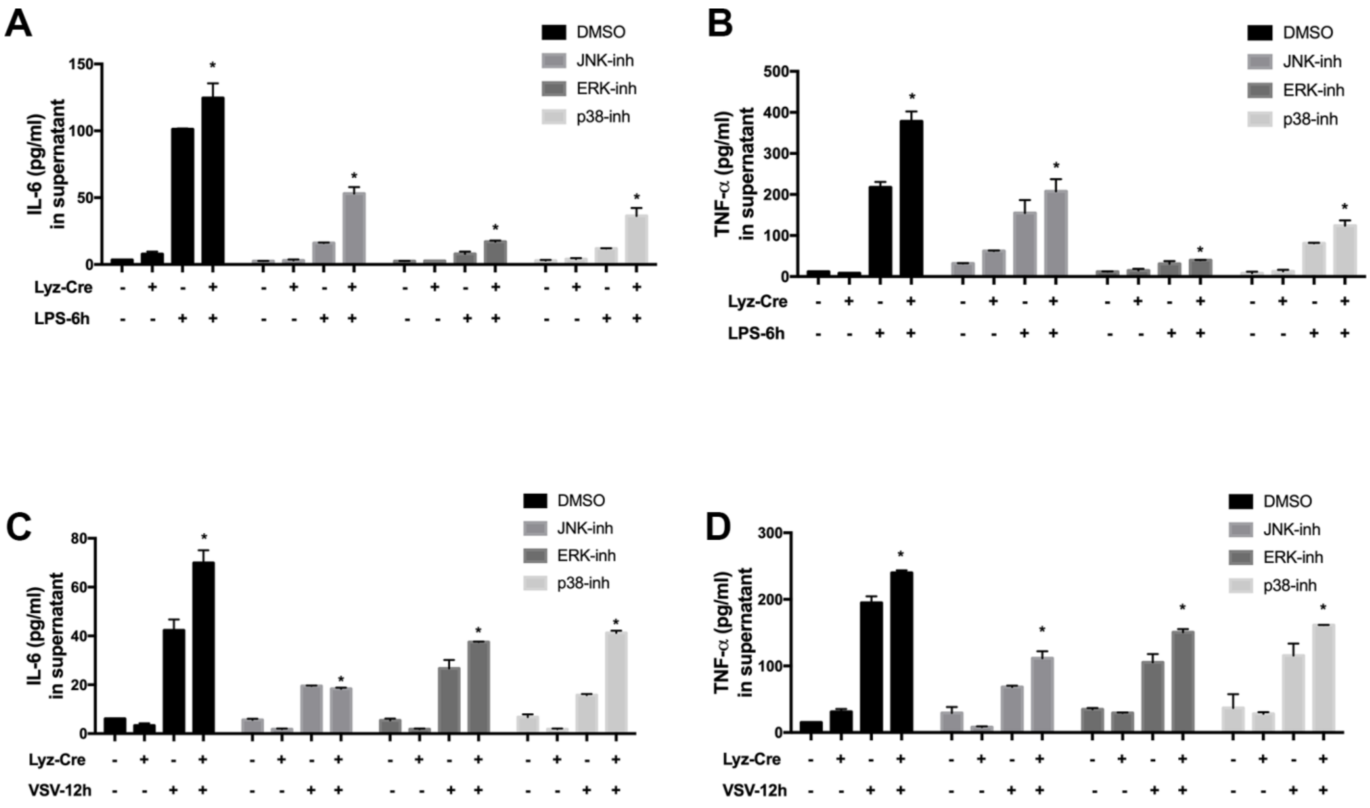

Supplementary Figure 5. (**A**–**C**) ELISA of IL-6 (**A**, **C**) and TNF-α (**B**, **D**) in the supernatant of peritoneal macrophage of Ets2$^{fl/fl}$Lyz2cre^{-} or Ets2$^{fl/fl}$Lyz2cre^{+} mice, pre-treated with DMSO or inhibitor of ERK, JNK, and p38 respectively and stimulated with 100 ng/ml LPS 6h (**A**, **B**) or 10 MOI VSV (**C**, **D**) for 12h. Data are shown as the mean ± s.d. of three samples. Student's t-test compared with the DMSO. *, P<0.05.

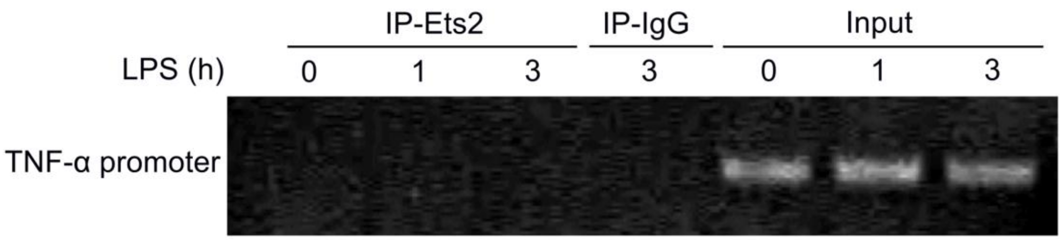

Supplementary Figure 6. ChIP analysis of the TNF-α promoter using an Ets2 antibody in mouse primary peritoneal macrophages treated with LPS for the indicated times.
